# Bedtime Procrastination, Sleep-Related Behaviors, and Demographic Factors in an Online Survey on a Polish Sample

**DOI:** 10.3389/fnins.2019.00963

**Published:** 2019-09-18

**Authors:** Radoslawa Herzog-Krzywoszanska, Lukasz Krzywoszanski

**Affiliations:** ^1^General Psychology Unit, Chair of Psychology, Faculty of Pedagogy, Pedagogical University of Kraków , Poland; ^2^Neurocognitive Psychology Unit, Chair of Psychology, Faculty of Pedagogy, Pedagogical University of Kraków , Poland

**Keywords:** health behaviors, bedtime procrastination, intention-behavior gap, sleep outcomes, sleep insufficiency, demographic factors, gender differences, students

## Abstract

The sufficient length and good quality of night sleep play a vital role in maintaining health, well-being and effective functioning. Nevertheless, an increase in the prevalence of sleep deprivation can be observed recently. The concept of bedtime procrastination, defined as going to bed later than intended, has been proposed to explain one of the psychological determinants of sleep deficiency. To investigate the prevalence of bedtime procrastination among Poles we carried out a Polish adaptation of the Bedtime Procrastination Scale (BPS), a self-report questionnaire for measuring the tendency to voluntarily postpone going to bed in the absence of any external circumstances for doing so. The aim of the research was to determine the main psychometric properties of the Polish version of the BPS. We also aimed to identify the relationships between bedtime procrastination and selected demographic variables in the Polish sample, and to examine the impact of bedtime procrastination on self-reported sleep outcomes. The data obtained from online surveys conducted on two Polish samples were analyzed, including demographic factors, self-reported sleep outcomes, and responses to items of the BPS. The Polish version of the BPS has a unifactorial structure like the original version. It also exhibits satisfactory internal consistency and moderate temporal stability in a 10-week retest study. BPS scores were not significantly related to the place of residence, the highest completed level of education, living with a spouse or partner, and living with children. Scores in BPS slightly decreased with age and females scored higher on BPS than males. Higher BPS scores were obtained for a group of students in comparison to a group of subjects who were not students, and lower BPS scores were found in working respondents in comparison to respondents who were not working. BPS scores correlate negatively with sleep length on workdays and a feeling of sleep sufficiency, and positively with sleep length on weekdays relative to workdays, sleeping later than one would like, and a feeling of fatigue. Several relationships between self-reported sleep outcomes and demographic variables were also identified.

## Introduction

Sleep is an important part of human life and is of key importance for physical and mental health, cognitive performance and good functioning at school, work, and leisure. Sleep deprivation may be the cause of poor working efficiency, low school performance (Wolfson and Carskadon, 2003; Curcio et al., 2006; Ming et al., 2011) traffic accidents (Connor et al., 2001; Connor, 2002; Abdoli et al., 2015a, b, 2018), mental stress, depressed mood and anxiety (Eller et al., 2006). It also involves medical conditions, including obesity, diabetes, cardiovascular disease, and an increased risk of death (Strine and Chapman, 2005; Gangwisch et al., 2006; Roane and Taylor, 2008; Fernandez-Mendoza et al., 2015). In addition, it was found that lack of sleep leads to a reduction in the level of optimism and sociability (Haack and Mullington, 2005).

Various factors may contribute to the delayed onset of night sleep. Length and quality of sleep are often negatively affected by various psychological and psychiatric issues such as insomnia and affective disorders, as well as several neurodegenerative diseases (Ondo et al., 2001; Bliwise, 2004; Herzog-Krzywoszanska and Krzywoszanski, 2019). Numerous studies have shown that female gender is a strong risk factor of poor sleep and insomnia; this is probably largely due to changes in sex steroid production during the menstrual cycle, pregnancy, and menopausal transition (Shaver et al., 1988; Pien and Schwab, 2004; Baker and Driver, 2007; Mong and Cusmano, 2016). On the other hand, numerous environmental and sociocultural factors, like exposure to noise and light at night, shift work, daily routines, lifestyle (Irish et al., 2015), and use of electronic media (Gradisar et al., 2013) all considerably contribute to postponing going to bed. Because of the growing mobility, accessibility, and user-friendliness of electronic media, we spend an increasing amount of time in front of screens (Rideout et al., 2010). As we devote more time to media, there is less time available for other activities, including sleep. One of the most profound effects of media use on sleep is sleep displacement, whereby media use leads to later bedtimes and shorter sleep duration (Cain and Gradisar, 2010; Van den Bulck, 2010).

The role of psychological conditions as the third potentially important group of factors in determining chronobiological health has been highlighted recently (Harvey, 2002; Harvey and Payne, 2002; Brand et al., 2015). Most research in this area focuses on patients with sleep disorders (ex., Szentkirályi et al., 2009; Swanson et al., 2011), but much less attention is paid to sleep problems that are a result of lifestyle and bad sleep habits in the general population (Kroese et al., 2016a). Going to bed on time may have a crucial role in providing sufficient sleep length and quality. Sleep deficiency can, therefore, be treated as the effect of a behavioral problem in that people have insufficient sleep because they go to bed late and the next morning they must get up for school or work. Most of them could fall asleep and sleep enough hours if they went to bed, but they delay doing so. Most often these individuals can easily predict that if they do not go to sleep early enough, they will be sleepy and tired the next day (Kroese et al., 2016b). Kroese et al. (2014, 2016a) called this phenomenon “bedtime procrastination,” defined as “needlessly and voluntarily delaying going to bed, despite foreseeably being worse off as a result” (Kroese et al., 2016b). Bedtime procrastination, like general procrastination, is associated with poor self-regulation. Self-regulation failure increases the tendency to seek immediate rewards, increases susceptibility to temptation and hinders concentration in goal-directed activities (Tangney et al., 2004). The ability to resist temptations is crucial in order to realize the intention to go to bed at a certain time by giving up attractive activities such as watching TV or surfing the internet (Hofmann et al., 2008; Mann et al., 2013). To measure the general tendency to go to bed later than intended, the Bedtime Procrastination Scale (BPS) was developed (Kroese et al., 2014). English (Kroese et al., 2014; Sirois et al., 2019), Danish (Kroese et al., 2016a) and Flemish (Exelmans and Van den Bulck, 2017) versions of the BPS have been used in previous studies on bedtime procrastination. In addition, the preliminary results of work on the Polish adaptation of BPS were presented recently by Herzog-Krzywoszanska and Krzywoszanski (2017).

In Poland, as in other countries, sleep deprivation is a fairly common problem (National Sleep Foundation, 2008; Oginska et al., 2014). The results of a study on a representative sample of Polish adults (Boguszewski, 2016) showed that half of Poles declared that they sleep for less than 6 h at least once a week, and 8% that they always sleep less than 6 h. Students are particularly vulnerable to sleep deprivation since they have worse health habits than subjects in other groups (Trockel et al., 2000; Buboltz et al., 2001; Gaultney, 2010; Owens et al., 2017). Research on Polish high-school students revealed that they were sleep-deprived as 88.5% of adolescents reported getting less than 9 h of sleep, and 78% adolescents felt tired during the day on 3 or more days per week (Kadzikowska-Wrzosek, 2018a). Nearly half of Polish university students always or often slept less than 6 h per day and over 60% felt tired in the morning (Błońska and Gotlib, 2012; Kasperczyk and Jośko, 2012). This high prevalence of sleep deprivation among students indicates the need for more comprehensive studies on the psychological factors that affect it. Taking into account the phenomenon of bedtime procrastination, which is recognized as a specific sleep-related deficit of self-regulation, this seems a very promising approach in this field. However, to the best of our knowledge no studies on the prevalence of bedtime procrastination in different demographic groups in Poland have yet been presented. In order to support our other aims, the first goal of our study was to develop a Polish version of the BPS and examine its psychometric properties. Secondly, since the role of demographic factors in bedtime procrastination has not yet been thoroughly analyzed, we intended to investigate the possible variations of BPS scores due to demographic variables. In particular, it seemed to us to be particularly interesting to compare the severity of bedtime procrastination in Polish students against subjects from groups with different professional status. Thirdly, we attempted to identify the impact of bedtime procrastination on sleep duration and sleep outcomes in the general Polish population.

## Materials and Methods

### Subjects

Since principal component analysis (PCA) and confirmatory factor analysis (CFA) had to be conducted on separate samples (Matsunaga, 2010), we conducted online surveys on two different samples of respondents. Sample 1 consisted of 431 students of the Pedagogical University of Kraków, who were studying various academic fields and disciplines. They participated voluntarily and received credit points for a voluntary academic course involving participation in research as subjects. Answers to survey questions were recorded anonymously. The possibility to withdraw from the research at any stage without providing explanations was assured. All students gave informed consent for their participation in the survey. Ten weeks (from 9 to 11 weeks) after the first test, the retest study was conducted on the participants from Sample 1.

Participants of Sample 2 were recruited via email invitations sent to a pool of research volunteers from the database maintained by Biostat, a Polish company providing an online social research service. For completing the survey participants were granted credit points with a value corresponding to five Polish zlotys (approximately 1 Euro). Credit points obtained by participants are redeemable for financial compensation from Biostat when a total sum of points worth at least 50 Polish zlotys has been accrued. Responses from 335 subjects from Sample 2 were analyzed as we excluded data from 42 participants (11.1% out of 377 respondents) who reported working nightshifts, indicated that they had received treatment for sleeping problems, or had consulted a doctor regarding sleep difficulties and were therefore considered as having a clinical history of sleep disorders. The minimum group sizes needed to detect the effect size of δ ≥ 0.5 with probability ≥ 0.8, assuming type I error rate two-tailed set at α = 0.05 with two-tailed testing were 295 and 36. The size of group 2 (students) relative to group 1 (other respondents) was assumed to be 0.12.

### Measures

The BPS is a self-report questionnaire consisting of nine individual items, that describe sleep-related behaviors and habits that are considered indicators of a high or low level of bedtime procrastination. The subject is asked to indicate whether given statements apply to him or her, choosing responses on a five-point Likert scale labeled 1 = “(almost) never” and 5 = “(almost) always.” Four items are reverse scored. The total BPS score is computed by averaging responses to all individual items and it may range from 1 to 5 points with a scale midpoint of 3 points. The total score reflects the extent to which people unnecessarily delay going to bed, with higher scores indicating more bedtime procrastination. Sample items are “I go to bed later than I had intended” and “I do not go to bed on time” (reverse coded). For the English version of BPS, Cronbach’s α of 0.92, 0.89, and 0.90 was obtained in an online survey of users of an internet crowdsourcing platform (Kroese et al., 2014) and on two samples of internet users (Sirois et al., 2019), respectively. Cronbach’s α of 0.88 was reported for the Dutch version of BPS in an online survey on a representative sample of Dutch adults (Kroese et al., 2016a) and for the Flemish version of BPS in a survey on a randomly selected sample of Flemish-speaking adults (Exelmans and Van den Bulck, 2017).

Demographic questions included gender, age, the place of residence (from village to big town/city), and education. The highest completed level of education (from lower secondary to doctoral, postgraduate, or equivalent) was coded according to the 2011 International Standard Classification of Education (ISCED) categories. Responses to questions about household composition and vocational status were also collected. For the purpose of data analysis, information about living with a child or children and living with a spouse or partner were extracted from the responses to the question about household composition and coded into two dummy variables with “yes” or “no” values. The responses to questions about vocational status were recoded into two dummy variables: employment (1 = employed, 0 = not employed) and student (1 = student, 0 = not a student).

Sleep descriptives and sleep outcomes were assessed using measures created by Kroese et al. (2014, 2016a). Participants’ self-perceived average night sleep duration was measured by answers to questions about sleep length on workdays and sleep length on weekdays; these were expressed in hours a day and given on the 5-point response scale (less than 5 | 5–6 | 7–8 | 9–10 | more than 10). The average frequency of going to bed later than intended was assessed by answers to the question “In an average week, how many days do you go to bed later than you would like to?,” given on the response scale described above. The answers to the question “On average, how many days a week do you feel tired during the day?” were given on a five-point response scale (0 days/never | 1–2 days | 3–4 days | 5–6 days | 7 days/always) and were used as a self-report measure of sleep-related daytime fatigue. Experienced sleep insufficiency was assessed by responses to the question “To what extent do you feel the number of hours of sleep you usually get is sufficient,” given on a four-point response scale (completely insufficient | rather insufficient | rather sufficient | completely sufficient).

### Procedure

The Polish BPS version was prepared in accordance with the rules of the translation and backtranslation procedure suggested by Brislin (1986). The initial translations of the BPS items were done by three Polish-speaking people (including two psychologists with Ph.D. in psychology) with a very good command of English. After comparing all Polish translations, the final Polish version was agreed through discussion (see: Appendix). The final Polish version of BPS items was subsequently back-translated into English, showing a satisfactory convergence with the original.

### Statistical Analysis

Principal component analysis that determined the number of components based on simulations on random data obtained in parallel analysis was performed as an exploratory assessment of the factorial structure of responses to items of the Polish BPS version in Sample 1. Bartlett’s test of the sphericity of the correlation matrix and the Kaiser-Meyer-Olkin measure of sampling adequacy (MSA) were also computed. To verify the unifactorial structure of the Polish version of BPS that was indicated by the results of PCA analysis, the responses obtained in Sample 2 were subjected to CFA. A single-factor model with one latent variable representing total BPS score was specified. Since the distribution of responses to items in the Polish BPS version deviated from normal distribution, the diagonally weighted least squares (DWLS) estimation method with robust error estimation was used. Chi-square to df ratio (χ^2^/df), root mean square error of approximation (RMSEA), standardized root mean square residual (SRMR), Bentler’s Comparative Fit Index (CFI), Tucker-Lewis Index (TLI), and Bentler-Bonett Normed Fit Index (NFI) were used to evaluate the overall model fit.

Cronbach’s α (Cronbach, 1951) and McDonald’s ω (McDonald, 1999) were applied as measures of the internal consistency of the total score in the Polish BPS version. Means, standard deviations, and item-rest correlations were computed. Measures of internal consistency were also calculated if an item was dropped for individual items. Pearson correlation was used for analysis of test–retest reliability. The R ‘cocron’ package for the statistical comparison of Cronbach’s α coefficients (Diedenhofen and Musch, 2016) was used to compare values of Cronbach’s α in independent samples. Values of standard error of measurement (Guilford, 1954; Harvill, 1991), the halfwidth of its 95% confidence interval, and minimal detectable change, also called smallest real change (Beckerman et al., 2001) were determined on the basis of Cronbach’s α as the measure of scale reliability. Mean, median, standard deviation, skewness, and kurtosis was given to describe the distribution of raw scores of the Polish BPS version in Sample 2.

Since the distribution of BPS scores were slightly skewed, the relations between raw total BPS scores and responses to sleep-related questions with demographic factors were examined using Spearman rank-order correlation and the Mann–Whitney *U*-test, with rank-biserial correlation as the measure of effect size. To find the best subset of demographic predictors of high severity of bedtime procrastination (defined as a high categorized score in BPS), multivariate binomial logistic regression was conducted to predict the ratio for high versus not-high BPS scores from demographic variables (gender, median-split dichotomized age, living with a spouse or partner, living with children, being a student and employment status). The backward elimination of variables with *p*-values > 0.05 in each step was applied.

A series of univariate ordinal logistic regression analyses predicting answers to sleep-related questions from raw BPS scores was performed to examine the impact of bedtime procrastination on sleep outcomes. To determine whether the impact of BPS scores on responses to demographic questions can be attributed to demographic variables, a series of two-step hierarchical multiple ordinal logistic regression analyses was performed in which demographics were entered in the first step, and sleep outcomes were entered in the second step.

The interrelationships between answers to sleep-related questions, respondents’ highest completed level of education, and the place of residence were analyzed using Spearman rank-order correlations. The impact on answers to sleep-related questions of gender, median-split dichotomized age (below 38 years versus 38 years or more), living with a spouse or partner, living with children, being a student and employment were examined using univariate ordinal logistic regressions. Multivariate ordinal logistic regressions with backward elimination of variables with *p*-values > 0.05 in each step were also conducted to select the best demographic predictors of sleep outcomes.

Jamovi (The jamovi project 2019) and JASP (JASP Team 2019) open-source statistical programs were used for statistical analyses. The lavaan R Package for Structural Equation Modeling (Rosseel, 2012) implemented in JASP was used for CFA computations.

## Results

### Demographic Characteristics of the Studied Samples

Distribution of respondents’ age and their responses to demographic questions are presented in Tables 1, 2.

**TABLE 1:** Distribution of respondents’ age in Sample 1 and Sample 2.

**Statistic**	**Sample 1**	**Sample 2**
Minimum	19	18
Lower quartile	20	28
Median	21	38
Upper quartile	23	49
Maximum	47	73
Mean	22.2	38.7
Standard deviation	3.23	13.3

**TABLE 2:** Frequencies of responses to demographic questions in Sample 1 and Sample 2 with percentages of total sample size.

**Variable**	**Category**	**Sample 1**	**Sample 2**
Gender	Female	383(88.9%)	171(51.0%)
	Male	48(11.1%)	164(49.0%)
Highest completed level of education	ISCED 2-24 Lower secondary – general	0(0.0%)	52(15.5%)
	ISCED 2-25 Lower secondary – vocational	0(0.0%)	55(16.4%)
	ISCED 3-34 Upper secondary – general	162(37.6%)	46(13.7%)
	ISCED 3-35 Upper secondary – vocational	32(7.4%)	27(8.1%)
	ISCED 4 or 5 Post-secondary or short cycle non-tertiary	128(29.7%)	30(9.0%)
	ISCED 6 Bachelor’s or equivalent	90(20.9%)	36(10.7%)
	ISCED 7 Master’s or equivalent	18(4.2%)	76(22.7%)
	ISCED 8 Doctoral or equivalent	1(0.2%)	13(3.9%)
Place of residence	Village	179(41.5%)	61(18.2%)
	Small town (below 50k inhabitants)	89(20.6%)	89(26.5%)
	Middle town (50 to 500k inhabitants)	40(9.3%)	109(32.5%)
	Big town/city (over 500k inhabitants)	123(28.5%)	76(22.7%)
Living with a spouse or partner	No	362(84.0%)	116(34.6%)
	Yes	69(16.0%)	219(65.4%)
Living with a child (children)	No	396(96.4%)	172(51.3%)
	Yes	15(3.6%)	163(48.7%)
Student	Not a student	0(0.0%)	292(87.2%)
	Student	431(100.0%)	43(12.8%)
Employment status	Not working		108(32.2%)
	Working		227(67.8%)

As can be seen in Tables 1, 2, Sample 2 represents a similar share of females and males and covers a wide range of ages and completed levels of education, with varied household composition; Sample 1 is dominated by young female adults with upper secondary or higher education, living without children and without a spouse or partner, all of which is due to their status as university students.

### Psychometric Characteristics of BPS

#### Principal Component Analysis

**FIGURE 1:**
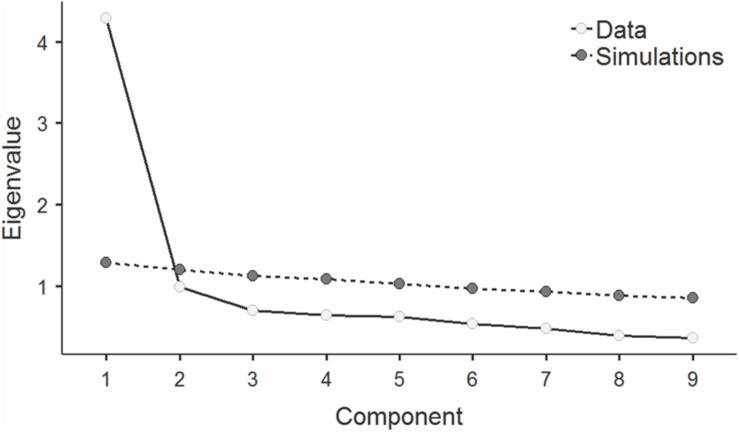
Scree plot with eigenvalues for consecutive components in principal component analysis (PCA) of responses to items of the Polish BPS version in Sample 1, with values of upper 95% confidence intervals for eigenvalues in simulations on random data obtained in parallel analysis.

Following the approach of the authors of the original BPS version (Kroese et al., 2014), PCA was used to explore the component structure of responses to items of the Polish version of this questionnaire in Sample 1 All interitem correlations of the Polish BPS version in this sample were significant at *p* < 0.001, ranging from 0.24 to 0.59, with average of 0.41. The results of Bartlett’s test of sphericity of the correlation matrix, χ*^2^*(36) = 1354, *p* < 0.001, showed significant relationships among responses to the individual items of the Polish version of BPS in Sample 1 and indicated that they are correlated enough for PCA. The overall Kaiser-Meyer-Olkin MSA was high (MSA = 0.90) and all MSA values for individual items were > 0.87, which confirmed the good factorability of responses to items of the Polish BPS version in this sample. Only the first eigenvalue was greater than one (4.29), and the inspection of a scree plot also suggested the extraction of the component which accounted for 48 percent of the total variance. Parallel analysis confirmed the adequacy of the one-dimensional solution (see Figure 1). Since only one component was extracted, no rotation was applied. As depicted in Table 3, all component loadings for PCA in Sample 1 were high, ranging from 0.60 to 0.79.

**TABLE 3:** Results of Kaiser-Meyer-Olkin measure of sampling adequacy (MSA), principal component analysis (PCA), and confirmatory factor analysis (CFA) of responses to items of the Polish BPS version.

	**Sample 1**			**Sample 2**	
		
**BPS item**	**Kaiser-Meyer -Olkin MSA**	**Component loadings in PCA**	**Uniqueness in PCA**	**Factor loadings in CFA**	**Standardized errors in CFA**
1	0.901	0.780	0.391	0.755	0.430
2	0.876	0.681	0.536	0.506	0.744
3	0.869	0.614	0.623	0.476	0.773
4	0.891	0.769	0.408	0.737	0.457
5	0.878	0.603	0.636	0.484	0.766
6	0.896	0.791	0.374	0.720	0.482
7	0.918	0.676	0.543	0.670	0.550
8	0.923	0.627	0.607	0.560	0.687
9	0.933	0.640	0.590	0.521	0.729

#### Confirmatory Factor Analysis

To verify the validity of the unifactorial structure of the Polish BPS version that were suggested by the results of PCA analysis, the responses obtained in Sample 2 were subjected to CFA. All path coefficients between the latent variable and individual BPS items were significant at *p* < 0.001, with standardized estimates (factor loadings) ranging from 0.48 to 0.76 (see Table 3 for details), thus indicating that the factor that represents the total BPS score substantially contributed to the variance of all BPS items. It is worth noting that the configuration of values of component loadings and uniqueness in PCA correspond to factor loadings and errors in CFA. The values of the adopted fit indices indicated a good overall fit of the specified CFA model: χ^2^/df = 1.31, RMSEA = 0.031, with 90% confidence intervals from 0.000 to 0.053, SRMR = 0.052, CFI = 0.994, TLI = 0.992, NFI = 0.976.

#### Items’ Descriptives, Item-Rest Correlations, and Scale Reliability

Means, standard deviations, item-rest correlations and measures of internal consistency if an item was dropped are given in Table 4 for individual items of the Polish BPS version in Sample 1 and Sample 2. Item-rest correlations in Sample 1 ranged from 0.50 to 0.70, with an average of 0.59; in Sample 2 they ranged from 0.44 to 0.67, with an average of 0.54.

**TABLE 4:** Means, standard deviations, item-rest correlations for BPS items and measures of internal consistency (Cronbach’s α and McDonald’s ω), computed if items dropped for Sample 1 and Sample 2.

**Sample**	**BPS item**	**Mean**	**Standard deviation**	**Item-rest correlations**	**Cronbach’s α**	**McDonald’s ω**
					
					**If item dropped**	
	1	3.78	1.26	0.688	0.835	0.838
	2	3.07	1.34	0.582	0.845	0.849
	3	2.83	1.36	0.511	0.852	0.855
	4	3.47	1.28	0.677	0.836	0.839
1.	5	3.11	1.32	0.495	0.853	0.856
	6	3.33	1.30	0.699	0.833	0.836
	7	1.99	1.23	0.571	0.846	0.849
	8	2.98	1.37	0.519	0.851	0.854
	9	2.59	1.27	0.539	0.849	0.853

	1	3.71	1.17	0.673	0.804	0.808
	2	3.28	1.34	0.461	0.826	0.832
	3	3.09	1.36	0.438	0.829	0.834
	4	3.29	1.30	0.665	0.803	0.809
2.	5	3.16	1.31	0.435	0.828	0.834
	6	3.43	1.30	0.643	0.806	0.811
	7	2.56	1.39	0.608	0.809	0.817
	8	3.13	1.34	0.501	0.821	0.827
	9	2.77	1.32	0.478	0.824	0.831

#### Reliability and Measurement Error of BPS Total Score

As presented in Table 5, Cronbach’s α and McDonald’s ω for the whole scale were greater than 0.8 in both samples. The values of Cronbach’s α did not differ significantly between both samples, χ^2^(1) = 2.01, *p* = 0.156, and the 95% confidence intervals of this coefficient overlapped considerably. Cronbach’s α for the Polish BPS version in Sample 2 differed significantly from Cronbach’s α obtained for the English (Kroese et al., 2014), Dutch (Kroese et al., 2016a), and Flemish (Exelmans and Van den Bulck, 2017) BPS versions: χ^2^(1) = 22.3, *p* < 0.001; χ^2^(1) = 13.4, *p* < 0.001; χ^2^(1) = 11.5, *p* = 0.001, respectively. It also differed significantly from Cronbach’s α for the English BPS version applied by Sirois et al. (2019) in study 1, χ^2^(1) = 6.03, *p* = 0.014, and study 2, χ^2^(1) = 23.6, *p* < 0.001. The Pearson correlation coefficient was equal to 0.675 in the 10-week test–retest analysis of total BPS score in 395 subjects with non-missing data from Sample 1, which indicates moderate temporal stability of the BPS score. Standard error of measurement values and halfwidth of the 95% confidence interval for measurement error for the total score of the Polish BPS version in Sample 2 are given in Table 5.

**TABLE 5:** Reliability statistics, standard error of measurement with halfwidth of 95% confidence intervals and minimal detectable change for the Polish BPS version.

**Statistic**	**Sample**	**Value of statistic (95% confidence intervals)**
		
Average interitem correlation	1	0.406
	2	0.361
Cronbach’s α	1	0.859 (0.839 | 0.878)
	2	0.834 (0.806 | 0.859)
McDonald’s ω	1	0.862
	2	0.839
Pearson correlation for test–retest	1	0.675 (0.618 | 0.726)
Standard error of measurement^∗^	2	0.351
Halfwidth of the 95% confidence interval for measurement error^∗^	2	0.668
Minimal detectable change^∗^	2	0.973

*^∗^Statistics computed with value of Cronbach’s α in Sample 2 as the measure of scale reliability. 95% confidence intervals for Cronbach’s α and Pearson correlation are given in parentheses.*

#### Distribution of Raw and Categorized BPS Total Score

Considering that Sample 2 was more diverse in terms of demographic variables, the results obtained in this sample were used to compute the measures of distribution of the raw total score in the Polish BPS version. The observed minimum and maximum are equal to the lowest and highest possible values of the scale (1 and 5 points), which shows that the obtained raw scores fully cover the scale’s entire range. Mean, median, standard deviation, skewness, kurtosis, and their 95% confidence intervals for Sample 1 and Sample 2 are presented in Table 6.

**TABLE 6:** Descriptive statistics with 95% confidence intervals where appropriate for distribution of raw total score of Polish PBS version in Sample 2.

**Statistic**	**Value of statistic (95% confidence intervals)**
	
Mean	3.224 (3.132 | 3.316)
Median	3.222 (3.111 | 3.333)
Std. dev.	0.861
Skewness	−0.358 (−0.619 | −0.097)
Kurtosis	−0.326 (−0.847 | 0.195)

*Confidence intervals are given where appropriate.*

**FIGURE 2:**
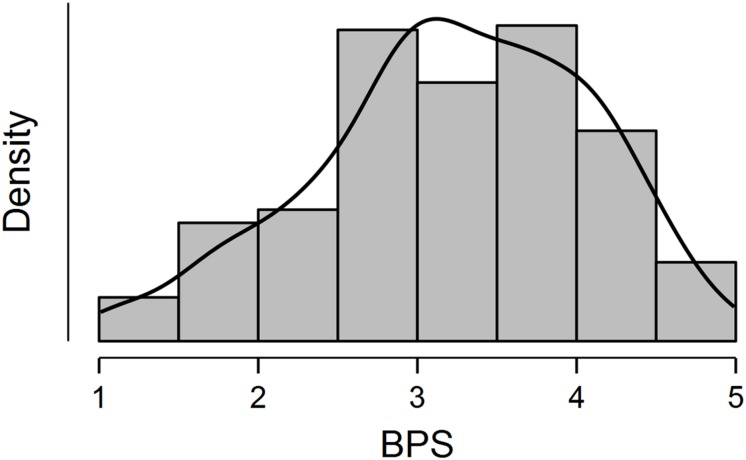
Histogram and smoothed density plot showing distribution of total raw scores of the Polish version of the Bedtime Procrastination Scale (BPS) in Sample 2.

Since both mean and median are higher than the scale midpoint (3.0) with a negative value of skewness, the distribution of the total scores of the Polish BPS version is slightly left-skewed. A histogram with a smoothed density plot showing the distribution of total scores of the Polish BPS version in Sample 2 is depicted in Figure 2.

**FIGURE 3:**
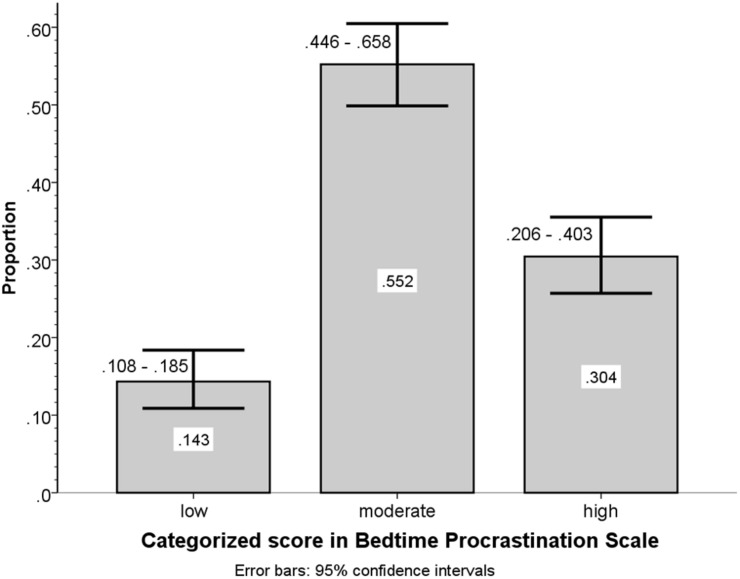
Group frequency to total sample size proportions with 95% confidence intervals for categorized scores of the Polish version of the BPS in Sample 2.

For discriminating between low, moderate and high levels of the Polish BPS version scores, two cut-off points that are distant by the halfwidth of the 95% confidence interval for the measurement error (Lyles and Kupper, 1999) from the scale midpoint were determined to have values of 2.332 and 3.668. Proportions of subjects with three levels of scores in the Polish BPS version are depicted in Figure 3.

### Relations Between BPS Scores and Demographic and Sleep-Related Variables

#### BPS Scores and Demographic Variables

**FIGURE 4:**
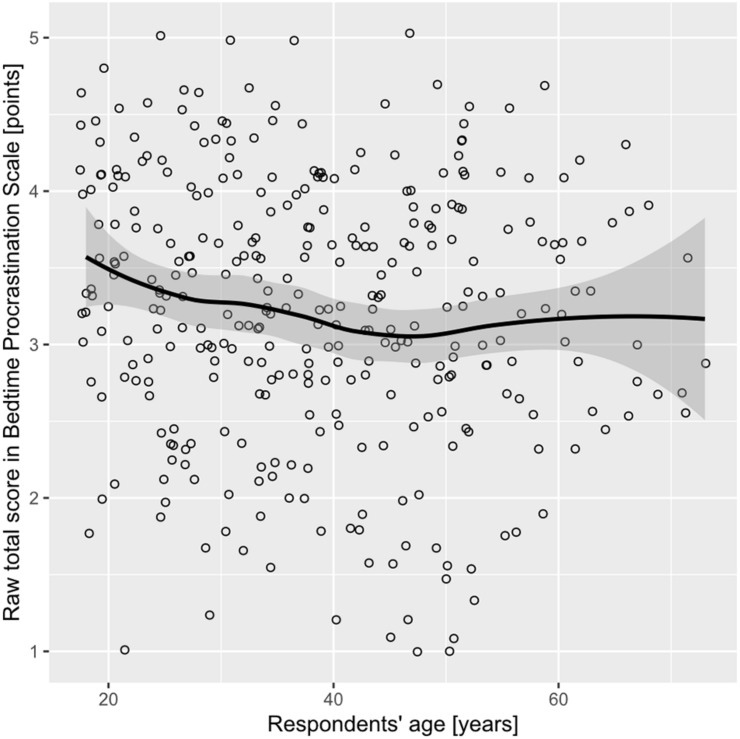
Scatterplot of raw total scores in BPS by respondents’ age with smoothed regression line and 95% confidence interval in Sample 2. The loess function was used for smoothing.

Spearman rank-order correlations between raw BPS scores and respondents’ age (ρ = −0.120 with 95% confidence intervals from −0.224 to −0.013) indicate that bedtime procrastination slightly decreases with age. The relationship between BPS scores and respondents’ age are plotted in Figure 4. As can be inferred from the confidence bands in Figure 4, mean BPS scores for respondents aged 18 to 37 deviate from the scale midpoint, whereas for subjects aged 38 or more years the scale midpoint is within the range of 95% confidence intervals (compare the confidence interval bands with the horizontal grid line at BPS value 3.0 in Figure 4).

Spearman rank-order correlations between raw BPS scores and respondents’ highest completed level of education (ρ = −0.101 with 95% confidence intervals from −0.206 to 0.006) and place of residence (ρ = 0.018 with 95% confidence intervals from −0.089 to 0.125) did not deviate significantly from zero. Results of the Mann–Whitney *U*-test that was used to examine the differences in raw BPS scores between groups distinguished by binomial demographic variables are presented in Table 7. The group means for raw BPS scores, probabilities and odds of high BPS categorized scores, and odds ratios for high versus not-high BPS scores are also given. Females scored higher on the BPS than males; higher BPS scores were obtained for the group of students in comparison to the group of non-students, and lower BPS scores were found in working respondents in comparison to non-working respondents.

**TABLE 7:** Comparison of total score of Polish PBS version for different categories of demographic variables in Sample 2.

**Variable**	**W (p)**	**Rank-biserial correlation (95% CI)**	**Category**	**BPS mean**	**Probability for high BPS score**	**Odd for high BPS score**	**Odds ratio for high/not high BPS score (95% CI)**
Gender	11718 (0.009)	0.164 (0.042 | 0.282)	Female	3.34	0.380	0.613	2.11 (1.30 | 3.40)
			Male	3.11	0.226	0.291	Reference
Spouse (partner)	11162 (0.068)	−0.121 (−0.247 | 0.008)	Living with a spouse (partner)	3.16	0.274	0.377	0.665 (0.411 | 1.08)
			Living without a spouse (partner)	3.34	0.362	0.568	Reference
Children	14374 (0.688)	0.025 (−0.098 | 0.148)	Living with children	3.25	0.294	0.417	0.912 (0.572 | 1.45)
			Living without children	3.20	0.314	0.458	Reference
Student	7736 (0.014)	0.232 (0.051 | 0.398)	Student	3.49	0.442	0.792	1.99 (1.04 | 3.83)
			Not a student	3.18	0.284	0.397	Reference
Employment status	9934 (0.005)	−0.190 (−0.313 | −0.059)	Working	3.13	0.269	0.367	0.600 (0.369 | 0.977)
			Not working	3.41	0.380	0.612	Reference

*W is the W statistic for the Mann–Whitney *U*-test; CI is confidence intervals. BPS high scores defined as BPS mean is greater than the 3.668 points cut-off. *p*-Values for given odds ratios are equal to *p*-values for the Mann–Whitney *U*-test.*

To find the best subset of demographic predictors of a BPS score above 3.668 points cut-off, multivariate binomial logistic regression was conducted to predict the ratio of high to not-high BPS scores from demographic variables, with backward elimination of variables with *p*-values > 0.05 in each step. Female gender and being a student were selected as best demographic predictors of high BPS scores (odds ratio: 2.17 with 95% confidence intervals from 1.31 to 3.44, and 2.04 with 95% confidence intervals from 1.05 to 3.96, respectively). The test of the overall model was significant (χ^2^ = 10.8 with 2 degrees of freedom, *p* = 0.004), and Nagelkerke pseudo *R*^2^ was equal to 0.057.

#### Scores in BPS and Responses to Sleep-Related Questions

Relationships between responses to questions about sleep duration and sleep outcomes, and the total score of the Polish PBS in Sample 2 are presented in Table 8. Raw total scores in BPS correlate negatively with sleep length on workdays and a feeling of sleep sufficiency, and positively with sleep length on weekdays relative to workdays, sleeping later than one would like and a feeling of fatigue.

**TABLE 8:** Responses to sleep-related questions and averaged raw scores in Polish BPS version with Spearman rank-order correlations in Sample 2.

**Question**	**Response category**	***n***	***n* to sample size ratio [%]**	**BPS mean**	**Spearman correlation (95% CI) *p***
Sleep length on workdays	Less than 5	10	3.0%	4.00	−0.339 (−0.430 | −0.240) <0.001
	5–6	117	34.9%	3.57	
	7–8	187	55.8%	2.98	
	9–10	21	6.3%	3.12	
	More than 10	0	0.0%		

Sleep length on weekdays	Less than 5	5	1.5%	3.62	−0.055 (−0.161 | −0.053) 0.320
	5–6	42	12.5%	3.50	
	7–8	164	49.0%	3.14	
	9–10	119	35.5%	3.20	
	More than 10	5	1.5%	3.96	

Sleep length on weekdays relative to workdays	Over 3 h shorter	1	0.3%	3.67	0.195 (0.089 | 0.296) <0.001
	1–3 h shorter	11	3.3%	3.08	
	Equal	151	45.1%	3.07	
	1–3 h longer	145	43.3%	3.28	
	Over 3 h longer	27	8.1%	3.80	

Sleep later than would like	Never	22	6.6%	1.87	0.416 (0.653 | 0.760) <0.001
	1–2 days	94	28.1%	2.58	
	3–4 days	100	29.9%	3.41	
	5–6 days	53	15.8	3.65	
	Always	66	19.7%	3.98	

Feeling of fatigue	Never	19	5.7%	2.64	0.338 (0.239 | 0.429) <0.001
	1–2 days	124	37.0%	2.95	
	3–4 days	96	28.7%	3.38	
	5–6 days	41	12.2%	3.38	
	Always	55	16.4%	3.66	

Feeling of sleep sufficiency	Completely insufficient	25	7.5%	3.58	−0.382 (−0.470 | −0.287) <0.001
	Rather insufficient	138	41.2%	3.53	
	Rather sufficient	148	44.2%	3.00	
	Completely sufficient	24	7.2%	2.44	

*CI, confidence intervals. Spearman rank-order correlation was computed for relationships between raw BPS scores and answers to sleep-related questions.*

The results from univariate and multivariate hierarchical ordinal logistic regression analyses that predict answers to sleep-related questions from raw BPS scores are presented in Table 9. Except for sleep length on weekdays, for all remaining sleep-related questions adding BPS scores to the regression models with demographics significantly improved the model’s fit. No considerable differences could be found when the values of pseudo *R*^2^ in univariate analyses were compared to pseudo *R*^2^ changes for adding BPS scores to the model in hierarchical analyses. This indicates that the impact of BPS scores on responses to sleep-related questions cannot be attributed to demographic variables.

**TABLE 9:** Odds ratios (with 95% confidence intervals) for ordinal logistic regressions predicting answers to sleep-related questions from raw BPS scores in univariate models and multivariate hierarchical models controlled for demographic variables in Sample 2.

	**Effects of BPS scores in univariate analyses**		**Effects of BPS scores controlled for demographic variables in multivariate analyses**		
		
**Sleep-related question**	**Nagelkerke pseudo *R*^2^**	**Odds ratio (95% CI)**	**Total model χ^2^ change (*p*)**	**Nagelkerke pseudo *R*^2^ change**	**Odds ratio (95% CI)**
Sleep length on workdays	0.070	0.456^∗∗∗^ (0.348 | 0.592)	36.0 (<0.001)	0.069	0.442^∗∗∗^ (0.333 | 0.582)
Sleep length on weekdays	0.001	0.908 (0.718 | 1.15)	1.07 (0.301)	0.002	0.879 (0.687 | 1.12)
Sleep length on weekdays relative to workdays	0.020	1.54^∗∗∗^ (1.22 | 1.97)	12.7 (<0.001)	0.020	1.57^∗∗∗^ (1.22| 2.02)
Sleep later than desired	0.291	9.93^∗∗∗^ (7.09 | 14.2)	230 (<0.001)	0.281	10.5^∗∗∗^ (7.39 | 15.6)
Feeling of fatigue	0.057	2.24^∗∗∗^ (1.75 | 2.88)	37.3 (<0.001)	0.050	2.18^∗∗∗^ (1.69 | 2.82)
Feeling of sleep sufficiency	0.095	0.372^∗∗∗^ (0.282 | 0.487)	53.9 (<0.001)	0.092	0.364^∗∗∗^ (0.273 | 0.481)

*^∗∗∗^*p* < 0.001; CI, confidence intervals. Nagelkerke pseudo *R*^2^ change is given for adding the effect of BPS to the regression model in the second step into the model with demographic variables (gender, dichotomized age, spouse/partner, children, student, employed) entered in the first step.*

#### Answers to Sleep-Related Questions and Demographic Variables

Spearman rank-order correlations indicated that answers to the question measuring the feeling of fatigue correlated negatively with the highest completed level of education, ρ = −0.131 with 95% confidence intervals from −0.235 to −0.024. No significant differences from zero were found for correlations between the place of residence and responses to any sleep-related questions.

The odds ratios with 95% confidence intervals obtained in univariate analyses predicting sleep outcomes from demographic variables are given in Table 10. In backward elimination multivariate analyses, being a student was the only predictor of sleep length on workdays and sleep length on weekdays relative to workdays; living with children was the only predictor for sleep length on weekdays; female gender was the only predictor for feeling of fatigue. The obtained odds ratios are identical to the corresponding ones from the univariate analyses (see Table 10). Living with children and employment were included in the final step (odds ratio: 1.70 with 95% confidence intervals from 1.15 to 2.52, and 0.571 with 95% confidence intervals from 0.375 to 0.866, respectively) for going to bed later than one likes.

**TABLE 10:** Odds ratios with 95% confidence intervals for univariate ordinal logistic regressions predicting answers to sleep-related questions from demographic variables in Sample 2.

	**Demographic variable**					
	
**Sleep-related question**	**Gender (female vs. male)**	**Age (<38 years vs. older)**	**Spouse (partner)**	**Children**	**Student**	**Employed**
Sleep length on workdays	1.08 (0.711 | 1.65)	1.14 (0.747 | 1.73)	1.09 (0.700 | 1.69)	0.920 (0.605 | 1.40)	0.482^∗^ (0.259 | 0.894)	1.17 (0.744 | 1.83)
Sleep length on weekdays	1.07 (0.714 | 1.61)	0.755 (0.502 | 1.13)	0.588^∗^ (0.381 | 0.903)	0.504^∗∗^ (0.332 | 0.760)	2.18^∗^ (1.15 | 4.18)	1.05 (0.676 | 1.63)
Sleep length on weekdays relative to workdays	1.04 (0.692 | 1.57)	0.724 (0.480 | 1.09)	0.593^∗^ (0.384 | 0.915)	0.608^∗^ (0.402 | 0.916)	3.69^∗∗∗^ (1.92 | 7.2)	1.01 (0.654 | 1.56)
Sleep later than would like	1.44 (0.983 | 2.12)	0.860 (0.586 | 1.26)	1.14 (0.768 | 1.7)	1.57^∗^ (1.07 | 2.31)	1.15 (0.673 | 1.98)	0.623^∗^ (0.412 | 0.940)
Feeling of fatigue	2.29^∗∗∗^ (1.55 | 3.42)	0.719 (0.487 | 1.06)	1.00 (0.667 | 1.5)	0.902 (0.612 | 1.33)	1.49 (0.842 | 2.62)	0.688 (0.454 | 1.04)
Feeling of sleep sufficiency	0.683 (0.454 | 1.02)	0.991 (0.661 | 1.48)	1.32 (0.862 | 2.03)	1.05 (0.702 | 1.58)	0.676 (0.373 | 1.22)	0.947 (0.615 | 1.46)

*^∗^*p* < 0.05, ^∗∗^*p* < 0.01, ^∗∗∗^*p* < 0.001, 95% confidence intervals are given in parentheses. Age was dichotomized into a binomial variable with 0 for less than 38 years and 1 for 38 years or more.*

## Discussion

In order to determine the psychometric properties of the Polish BPS version, we first tested whether its total score allows measurement of the general tendency to procrastinate going to bed, conceptualized as a uniform dispositional construct. Results of PCA on Sample 1 and CFA on Sample 2 consistently and clearly showed that responses to all individual items of the Polish BPS version are considerably intercorrelated and a substantial part of their variance could be attributed to one common latent variable: total BPS score. This justified computing the composite score of the Polish BPS version by averaging the values assigned to the responses to all individual items. All values of corrected item-rest correlations between responses to all individual items and total BPS score well above the recommended 0.3 threshold, showing that in both samples all the items were substantially related to the total scores computed from all other BPS items. The values of Cronbach’s α and McDonald’s ω for the whole scale were greater than 0.8 in both samples. It should also be noted that the values of Cronbach’s α obtained for the English (Kroese et al., 2014; Sirois et al., 2019), Dutch (Kroese et al., 2016a), and Flemish (Exelmans and Van den Bulck, 2017) BPS versions were slightly higher than Cronbach’s α for the Polish BPS version in Sample 2, which was drawn from the general population. However, the values of the internal consistency indicators obtained in our samples for the Polish BPS version are high enough to allow reliable qualitative measurement of the general tendency to procrastinate going to bed with a relatively low error of measurement. The use of the Polish BPS version also makes it possible to differentiate between subjects with varying levels of this behavioral tendency.

We also proposed two cut-off points for discriminating low, middle and high levels of bedtime procrastination, based on determining values distant from the scale midpoint by the halfwidth of the 95% confidence interval for the measurement error. By applying the given cut-off points, the level of bedtime procrastination of about one-third of respondents recruited from the general population were classified as high, whereas about half as many subjects demonstrated a low level of bedtime procrastination. The slightly left-skewed distribution of the total score of the Polish BPS version may be related to a possible ceiling effect which limits its suitability for differentiation between subjects with very high severity of bedtime procrastination. In further studies, this limitation could possibly be overcome by the extending the response format to a Likert scale with more than five points and modifying the anchor labels if necessary, in surveys conducted on subjects with very high bedtime procrastination. Moderate correlation in test–retest comparisons of BPS scores indicates relatively temporal stability of bedtime procrastination and suggests its dispositional status, but on the other hand it shows that it can be partially subject to change.

We found that in the Polish sample the level of bedtime procrastination was significantly higher by about half the standard deviation compared to international English-speaking users of an internet crowdsourcing platform (Kroese et al., 2014), a representative sample of Dutch adults participating in internet surveys (Kroese et al., 2016a) and a randomly selected sample of adults residing in Flanders, Belgium (Exelmans and Van den Bulck, 2017). In view of the similarity of our research methodology to the methodology of previous studies (Kroese et al., 2014, 2016a; Exelmans and Van den Bulck, 2017), there are no grounds for attributing the higher level of bedtime procrastination among Poles to any methodological issues. On the other hand, the mean BPS scores in the Polish sample do not differ substantially from the results reported by Sirois et al. (2019) for study 1 and study 2: *M* = 3.23 ± 0.89, and *M* = 3.05 ± 0.90, respectively. Further research on the role of socioeconomic and psychological factors in bedtime and sleep-related behaviors is needed to explain the relatively high level of bedtime procrastination found in the Polish sample, which was drawn from the general population.

Average BPS scores were related to reduced sleep length on workdays, increased sleep length on weekdays relative to workdays, a feeling of sleep insufficiency, and a feeling of fatigue. Despite the fact that responses to sleep-related questions showed several relationships with demographic variables, the results of multivariate analyses indicate that the impact of bedtime procrastination on self-reported sleep outcomes cannot be attributed to demographic variables.

In this study, we also attempted to delineate more precisely the differences in bedtime procrastination between demographic groups. We found a relatively low negative correlation between BPS scores and age (ρ = −0.120). A decrease in the mean level of bedtime procrastination with age was also reported several times in the results of previous studies. Significantly different-from-zero negative correlation coefficients between BPS scores and age were found by Exelmans and Van den Bulck (2017) in a sample of inhabitants of Flanders (*r* = −0.404), and by Sirois et al. (2019) in a sample of internet users (*r* = −0.32). A negative correlation between BPS scores and age (*r* = −0.11) was also obtained in a sample of users of an internet crowdsourcing platform (Kroese et al., 2014), but due to the relatively low sample size it did not meet the criterion of statistical significance. In addition, a negative correlation (*r* = −0.19) between age and bedtime procrastination assessed by means of sleep diaries was found in employees working in various industries (Kühnel et al., 2018). Apart from confirming a negative correlation between BPS scores and age, we also managed to capture the more detailed pattern of the associations between bedtime procrastination and age. The highest average BPS scores were observed in the group of the youngest respondents, with a weak decreasing tendency for the middle-aged group, followed by a relative stabilization of the scores around the scale midpoint in older participants (see Figure 4).

Place of residence, highest completed level of education, living with a spouse or partner, and living with children were not significantly associated with BPS scores. On raw BPS scores, females scored about one-fourth of the standard deviation higher than males. Higher BPS scores were obtained for a group of students in comparison to a group of non-student subjects, and lower BPS scores were found in working respondents in comparison to non-working respondents. Female gender and being a student appeared to be the best demographic predictors of high BPS scores in the analysis with backward elimination of parameters in the multivariate regression model, therefore the effects of age and employment could be explained by the impact of being a student because being young and unemployed considerably overlaps with being a student.

Although females scored slightly higher than males in the raw BPS scores, when taking into account the odds ratio for the effect of gender on categorized BPS scores (OR = 2.11 in univariate analysis, and OR = 2.17 in multivariate analysis) it should be noted that the chance of severe bedtime procrastination is more than twice as high for females than for males. Differences between the sexes in delaying sleep already appear in school children. Kubiszewski′s et al. (2014) research has shown that 70% of girls and only 55% of boys among adolescents aged 11–15 go to sleep after 10 p.m. The earlier age of onset of bedtime delay in girls than in boys may increase the risk of formation of poor sleep habits, thus leading to sleep deprivation. This problem is all the more important due to the fact that women report a greater need for sleep than men (Oginska and Pokorski, 2006; Cheng et al., 2012) and they also have a higher risk than men of developing cardiovascular and metabolic disease (Cappuccio et al., 2007; Ferrie et al., 2007; Suarez, 2008; Kronholm et al., 2011; Lyytikäinen et al., 2011; Miller, 2015) and depression (Armitage and Hoffmann, 2001; Krystal, 2004) as a result of sleep deprivation. A higher prevalence of sleep disorders and poorer sleep quality in women compared to men have been reported in several studies and attributed to biological factors (Manber and Armitage, 1999; Zhang et al., 2011, 2014; Nowakowski et al., 2013). Our research suggests that, along with biological factors, sleep-related behaviors, i.e., voluntarily delaying going to sleep without valid external reasons, could also be an important determinant of the poorer sleep quality in women.

The results of our research for the first time show that students have a higher level of bedtime procrastination than non-students. As can be inferred from effect of being a student on categorized BPS scores (OR = 1.99 in univariate analysis, and OR = 2.04 in multivariate analysis), the chance of severe bedtime procrastination is about twice as high for a group of students than for subjects who are not students. These findings are in line with much research that reports a considerable degree of sleep problems and poor sleep quality in students (Kang and Chen, 2009; Cheng et al., 2012). There are several possible reasons for the occurrence of sleep problems in students, including anxiety and depression (Moo-Estrella et al., 2005; Eller et al., 2006). However, as our research shows, an important reason for poor sleep quality in students may also be inappropriate sleep behavior. Students generally have poor health habits, including irregular meal patterns and high consumption of recreational drugs, both of which can impair sleep hygiene (Brown et al., 2002; Brick et al., 2010; Spanos and Hankey, 2010). Previous researchers have shown a high incidence of irregular sleeping habits and insufficient sleep in students (Kang and Chen, 2009). The research of Manber et al. (1996) revealed that regularizing sleep-wake schedules in students with irregular sleep schedules and excessive daytime sleepiness resulted in increased sleep efficiency and improved alertness compared to the control group. The lack of a regular sleep-wake schedule requires more attentional control and effort to regulate sleep behavior, which may be difficult, especially in the evening when self-regulatory resources are exhausted (Hofmann et al., 2012). Self-control allows delay of gratification in order to achieve a long-term goal, so it is crucial for the implementation of health intentions related to diet, physical activity or sleep. A number of studies have shown that self-control is negatively associated with bedtime procrastination (Kroese et al., 2014, 2016a; Exelmans and Van den Bulck, 2017; Kadzikowska-Wrzosek, 2018b). Self-control is important for behavior that allows you to go to sleep at the right time to get enough sleep; for example, refraining from drinking caffeinated beverages in the evening or engaging in raising the level of stimulation by watching exciting movies (Nauts and Kroese, 2017). Studies show that most bedtime procrastinators’ habitual leisure activities, such as watching TV or using other electronic media, can cause sleep delay (Kroese et al., 2014). Exelmans and Van den Bulck (2017) found positive correlations between evening television viewing and bedtime procrastination and between bedtime procrastination and deficient self-regulation of television viewing. These results suggest that low self-regulation makes it difficult to stop using electronic media when going to sleep, which leads to delayed sleep. Having good sleep habits, including regular bedtimes and waketimes, reduces the risk of self-regulation failure, especially in situations in which it is weakened due to tiredness when going to bed at night. Habit formation can be supported by self-regulation techniques such as intention implementation. Loft and Cameron (2013) found that the implementation intention intervention can improve sleep behavior and in turn improve sleep quality in workers. The effectiveness of the implementation intention and other interventions that normalize the rhythm of the day in students – thus forming proper sleep habits and preventing bedtime delay – requires further research.

## Conclusion

The Polish BPS version has psychometric properties similar to the original version. It allows reliable measurement of bedtime procrastination, conceptualized as a uniform construct. The level of bedtime procrastination among Poles is highly varied, with high scores clearly being much more common than low ones. Average BPS scores were related to worse self-reported sleep outcomes. In the youngest subjects, the highest averaged BPS scores were observed with a slightly decreasing tendency and subsequent stabilization around the scale midpoint in middle-aged and older respondents. Average total BPS scores were also dependent on the employment status of respondents. However, female gender and being a student were found to be the best demographic predictors of high BPS scores in the multivariate regression model, which indicates that the effects of age and employment status on bedtime procrastination stems from the fact, that students are younger and usually are not employed. Students should be considered as the group most vulnerable to bedtime procrastination; thus, they may require further research, as well as health promotion interventions to correct their sleep-related habits and attitudes.

## Data Availability

The data supporting the findings of this study will be made available by the authors to qualified researchers upon reasonable request.

## Ethics Statement

The studies involving human participants were reviewed and approved by the Faculty Committee for Research Ethics, Faculty of Pedagogy, Pedagogical University of Kraków. The patients/participants provided their written informed consent to participate in this study.

## Author Contributions

RH-K and LK contributed to the concept and design of the study, administration of the surveys, and revision of the manuscript, interpreted the results, wrote the sections of the manuscript, and read and approved the submitted version. LK designed and maintained the database, and performed the statistical analysis.

## Conflict of Interest Statement

The authors declare that the research was conducted in the absence of any commercial or financial relationships that could be construed as a potential conflict of interest.

## Appendix

### Bedtime Procrastinationscale – Polish Version

W odniesieniu do każdego z poniższych stwierdzeń zdecyduj w jakim stopniu dotyczy ono ciebie, wybierając odpowiedź na skali od 1 (nigdy lub prawie nigdy) do 5 (zawsze lub prawie zawsze):

1. Chodzę spać później niż zamierzałem(am).
2. Kładę się spać wcześnie, jeśli muszę wstać wcześnie rano (reverse coded).
3. Gdy wieczorem nadchodzi czas gaszenia światła, robię to od razu (reverse coded).
4. Często wciąż robię inne rzeczy, kiedy jest już czas żeby pójść spać.
5. Łatwo rozpraszają mnie różne rzeczy, kiedy właściwie już chciał(a)bym iść spać.
6. Nie chodzę spać na czas.
7. Mam stałą porę kładzenia się spać, której się trzymam (reverse coded).
8. Chcę iść spać o właściwej porze, ale po prostu nie mogę.
9. Łatwo mogę przerwać to co robię, kiedy jest czas kłaść się spać (reverse coded).
